# Network hubs cease to be influential in the presence of low levels of advertising

**DOI:** 10.1073/pnas.2013391118

**Published:** 2021-02-12

**Authors:** Gabriel Rossman, Jacob C. Fisher

**Affiliations:** ^a^Department of Sociology, University of California, Los Angeles, CA 90095;; ^b^Institute for Social Research, University of Michigan, Ann Arbor, MI 48104;; ^c^Social Science Research Institute, Duke University, Durham, NC 27708

**Keywords:** networks, diffusion, marketing, centrality, opinion leader

## Abstract

Attempts to find central “influencers,” “opinion leaders,” “hubs,” “optimal seeds,” or other important people who can hasten or slow diffusion or social contagion has long been a major research question in network science. We demonstrate that opinion leadership occurs only under conventional but implausible scope conditions. We demonstrate that a highly central node is a more effective seed for diffusion than a random node if nodes can only learn via the network. However, actors are also subject to external influences such as mass media and advertising. We find that diffusion is noticeably faster when it begins with a high centrality node, but that this advantage only occurs in the region of parameter space where external influence is constrained to zero and collapses catastrophically even at minimal levels of external influence. Importantly, nearly all prior agent-based research on choosing a seed or seeds implicitly occurs in the network influence only region of parameter space. We demonstrate this effect using preferential attachment, small world, and several empirical networks. These networks vary in how large the baseline opinion leadership effect is, but in all of them it collapses with the introduction of external influence. This implies that, in marketing and public health, advertising broadly may be underrated as a strategy for promoting network-based diffusion.

## Significance

A major focus of social network analysis is attempting to find central “influencers” or “opinion leaders” who can hasten or slow the spread of a social contagion. Using a simulation, we demonstrate that the most central node is important only under conventional but implausible scope conditions. We model the introduction of mass media or advertising and show that this allows social contagions to spread equally fast whether or not the seed node is highly central to the network. The most central node loses its relative importance even if mass media or advertising influence is extremely weak. This implies that, rather than targeting a node with a highly central position, marketers and public health officials should advertise broadly.

Among the central theoretical and practical attractions of social network analysis is the promise that key nodes, known as “opinion leaders” or “influentials,” hold structural power to change the ideas and behaviors of entire social systems (1–3). An extensive literature in sociology, physics, and network science centers on how best to measure network centrality. From the beginning, much of this literature takes as its motivation identifying a node or nodes that are optimal seeds for diffusion (4–8).* For instance, a seminal study of how doctors prescribe new drugs ascribed this behavior to key doctors in the advice network (11). In such applied contexts as “viral” marketing and public health outreach, opinion leadership suggests the promise that a structurally important node (and, by extension, the social network analyst who can identify that node) is the key to controlling the spread of a product, health behavior, or other idea or behavior (2, 3, 8, 12–14).

The influentials literature focuses on network sources of information, but in most realistic scenarios people have sources of information that transcend the network (15–17). Introducing these nonnetwork sources of information may qualitatively change the nature of diffusion, and specifically the role of a highly central hub or hubs. In many theories and simulations, agents are constrained to only observe information through a social graph, but real people are not so myopic. Even if we are most attentive to word of mouth from our social ties, we also learn about new ideas and behaviors from mass media, advertising, government mandates, and even direct observation of events. If it begins raining and everyone opens her umbrella, the proximate cause of this behavior is a response to nature rather than information spreading through a social network (18). Some diffusion models meaningfully incorporate roles for external sources of information (15, 19, 20), but other models effectively assume an entirely word-of-mouth process even if their narrative theory allows for external influence (3).

The computational experiment we present in this article contributes to a large body of social networks literature on influentials and opinion leadership (7, 8), but takes as its microfoundations a diffusion model from marketing that involves both network-based diffusion and external influence from sources like advertising (15). We conduct a large-scale computer simulation in which we seed diffusion with either the most central node or a node chosen at random in various empirical and algorithmically generated networks.^†^ We test the opinion leadership hypothesis for various points in parameter space where one axis is the strength of network-based diffusion (e.g., “word of mouth”) and the other axis is the strength of an external force (e.g., advertising and mass media). We measure the strength of opinion leadership for each point in parameter space by how much faster diffusion occurs when the initial node is highly central versus chosen at random.

The experiment adapts a mixed-influence model outlined by Bass (15, 21–23) to test whether the effect of central nodes on diffusion is robust to the presence of external influence. In the Bass model, people are exposed to information about the innovation from two sources: interpersonal imitation (with a density-dependent hazard) and external influence (with a constant hazard). Interpersonal influence represents the effect of word of mouth (or closely analogous processes like local network externalities or person-to-person spread) (24, 25). External influence represents the effect of advertising, mass media, internet search, or government mandates (15, 17, 23, 26). Traditionally, the Bass model is represented as a differential equation that measures diffusion in aggregate over time. The aggregate approach has the advantage of simplicity but makes it impossible to integrate network structure. We therefore adapt the Bass model to an agent-based model, which allows for potential emergent properties of unequal influence between nodes based on their structural positions.

The Bass model defines the rate of new adoptions in aggregate as follows:

$$\Delta N_{t}=\left(a+bN_{t}\right)\left(N_{\max}-N_{t}\right),$$

where $N_{t}$ is the cumulative number of people who have adopted as of time *t*, *a* is the coefficient of external influence, *b* is the coefficient of interpersonal influence, and $N_{\max}$ is the asymptotic number of people who will ever adopt. To include the effect of network structure on individual adoption, we adapt this equation to an agent-based model. In the agent-based model, for each agent *i* at time *t*:

$$p\left(i\mathrm{adopts}\mathrm{at}\mathrm{time}t|i\mathrm{has}\mathrm{not}\mathrm{adopted}\mathrm{before}t\right)=\alpha +\beta \left(\mathrm{fraction}\mathrm{of}i\text{'}s\mathrm{neighbors}\mathrm{who}\mathrm{adopted}\mathrm{before}\mathrm{time}t\right).$$

*α* is a constant hazard of adoption, representing the weight given to advertising and other external influences on diffusion, and *β* is the weight given to social or network influence.^‡^ To ensure that *α* and *β* are on comparable scales, we allow them to range between 0 and a maximum value that saturates the network with a consistent probability. We identify these maxima with a separate set of simulations, which identify the values at which *α* and *β* saturate the network in 100 ticks or less in 50% of trials. We refer to these *α* and *β* maxima as “LD50,” as a metaphor for the standard “lethal dose 50%” metric in toxicology. Full details of estimating the LD50 values appear in *SI Appendix*, *Determining parameter range*.§ To highlight changes at the lowest end of parameter space, we explore both dimensions of the parameter space on a log scale. In both the aggregate and agent-based Bass models, once a person adopts, she cannot abandon the innovation, meaning the number of adopters increases monotonically.

**Fig. 1.**
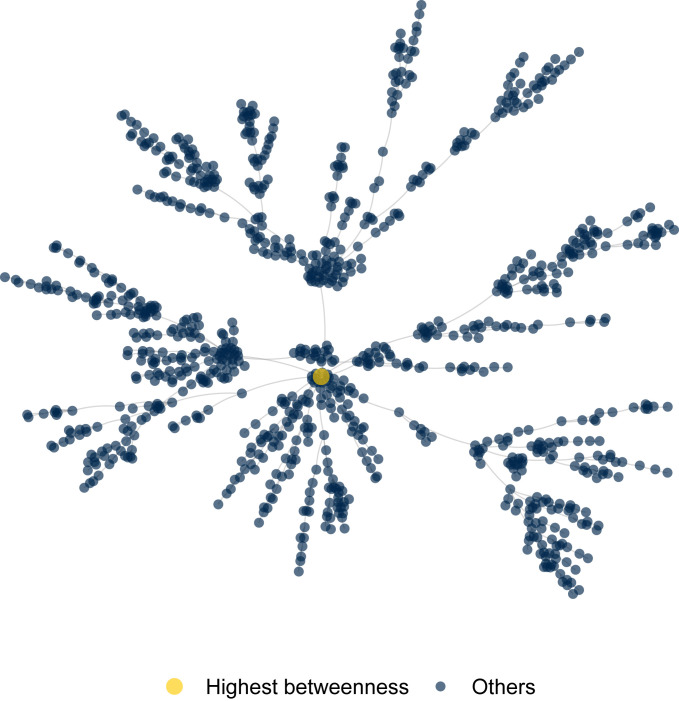
Example of a preferential attachment network generated with the Barabási–Albert algorithm (30) with 1,000 nodes, one edge per node, and an exponent of 1. We focus on this network as relatively favorable to opinion leadership but in Fig. 3 and *SI Appendix* show other networks. The yellow node is the highest betweenness node used to test the effect of influentials.

**Fig. 2.**
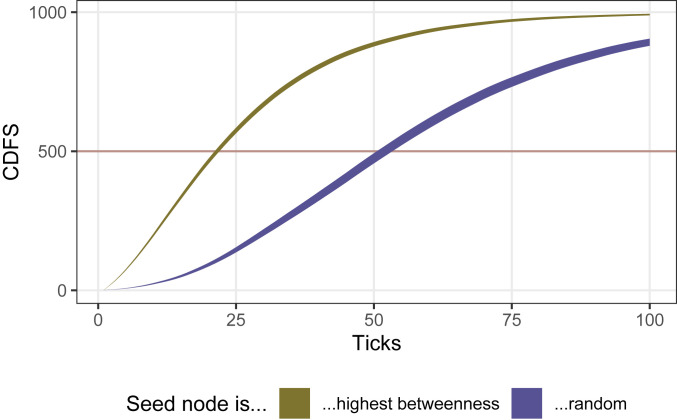
Cumulative number of adopters, denoted CDF, in simulations assuming only network diffusion (*α* = 0, *β* = LD50) in a preferential attachment network (1,000 nodes, one edge per node). The plot shows the confidence interval around the mean of both experimental conditions: simulations seeded with the highest betweenness node and simulations seeded with a randomly selected node. Seeding with the highest betweenness person saturates half the network (indicated by the red horizontal line) over twice as fast.

**Fig. 3.**
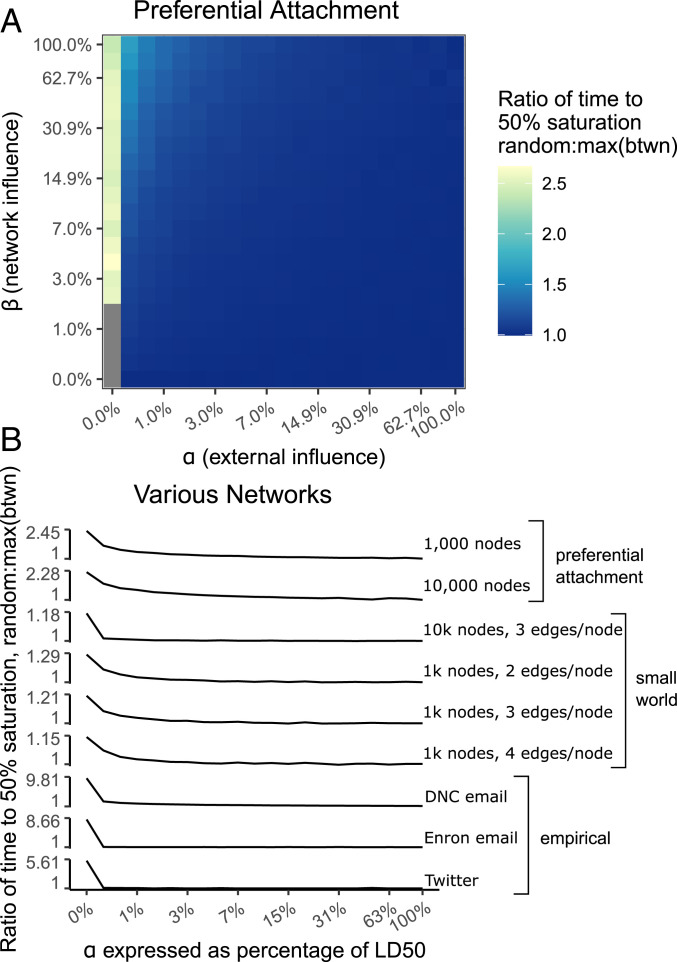
Ratio of mean time to midsaturation in simulations targeting a randomly chosen node in the network versus targeting the highest betweenness node. *A* shows the full parameter space for randomly generated preferential attachment networks (1,000 nodes, one edge per node). The gray cells represent right censored cases. Targeting a highly central person results in adoption that is over twice as fast, but only when there is no effect of advertising (α=0). *B* shows a summary across several algorithmically generated and empirical networks as we assume high levels of network diffusion (*β* = LD50) but vary external influence (α) as a percentage of each LD50 value, plotted on a logarithmic scale. This is the equivalent to the top row of cells in *A*, but substituting a *y* axis for the heat dimension and showing more networks. Across all these networks, targeting a highly central person results in faster adoption, but only when there is no effect of advertising (α=0). The impact of highly central seeds approaches parity with random seeds at even very low positive levels of advertising.

Our experimental setup varies the seed, meaning the initial innovator in the simulation. In the simulation, innovations start at one person, the seed, and spread outward from that person.^¶^ Our control condition seeds the innovation with a randomly chosen person in the network. Our treatment condition seeds the innovation with the most central person in the network, as measured by betweenness. In most networks, betweenness is right-skewed so in our networks the most central node is anywhere from six to several hundred SDs above the mean.^#^ We test the effects on preferential attachment networks (shown in Fig. 1) and small world networks generated in igraph (27) as well as the giant components of the Democratic National Committee email network (548 nodes and 2,442 edges), Enron email network (33,696 nodes and 180,811 edges), and a network of retweets and mentions on Twitter (532,325 nodes and 694,606 edges). We focus on preferential attachment networks in Figs. 1 and 2 but show robustness of our key finding to all these networks in Fig. 3 and *SI Appendix*.

Fig. 2 shows the central tendencies of the cumulative distribution functions by random versus highest betweenness seed node given the assumption of peak social influence (β = 0, β = LD50).^||^ Under those conditions, innovations that start with the most central person spread to half of the people in the network over twice as fast.

As Fig. 2 indicates, the gap between the conditions is approximately widest at time to 50% adoption (cumulative distribution function [CDF] = 500, displayed as a red horizontal line), making it the metric most favorable to opinion leadership. In addition, time to 50% adoption is much less vulnerable to right censorship than time to saturation. We use this metric, average time to 50% adoption, to summarize the full parameter space. In Fig. 3*A*, we demonstrate how diffusion speed on a preferential attachment network responds to varying the *α* and *β* parameters separately for random seeds and seeding at the highest betweenness node. The heat dimension shows the ratio of the mean time to 50% saturation for a random seed over that for a high centrality seed. (*SI Appendix*, Fig. S1 shows how this ratio is derived from Fig. 2.) Seeding with a highly central node has an advantage but only when *α* = 0. This advantage disappears quickly for all points in parameter space where *α* > 0, dropping precipitously at the next interval (*α* = 0.26% of LD50), and the advantage of a highly central seed node almost completely vanishes for points in parameter space where *α* > 3% of LD50.

The heat map in Fig. 3*A* only illustrates results for preferential attachment networks, but in Fig. 3*B* we provide sparklines summarizing several networks for the plane of parameter space where *β* = LD50 (i.e., the equivalent of the top row of the heat map). When *α* = 0, the effect of a highly central seed node varies substantially by the type of network, being trivial in a small world, but substantial in the three empirical networks. However, the finding from preferential attachment networks that the advantage of seeding with the peak betweenness node collapses rapidly when *α* > 0 replicates in all other networks, no matter how strong the highly central seed node effect is when α=0. Targeting the central node materially speeds adoption only in the region of parameter space where there is no external influence (*α* = 0). In all networks, there is a precipitous drop in the effect of highly central seeding as *α* goes from zero to 0.26% of the LD50 and the highly central seed effect is essentially absent when *α* reaches even a few percentage points of its LD50 value. *SI Appendix*, Figs. S3–S6 contains full heat maps for all networks listed in the sparklines plot of Fig. 3.

These findings indicate that the positive effect of targeting the most central node as opinion leader is subject to a highly restrictive scope condition. Previous research has shown that opinion leadership requires substantial inequality in centrality (28), but many phenomena of interest meet that scope condition. Here, we show the much more demanding scope condition of the absence of advertising or other forms of external influence. When no external influences are present, targeting a highly central person results in diffusion that can spread to half of the network faster than if a person were chosen at random, with the advantage being trivial for small world networks and an order of magnitude for the email networks. However, in the presence of external influences, even extremely weak external influences, identifying and seeding with an opinion leader do not lead to appreciably faster adoption of an innovation. This suggests that the simulation literature on optimal seeding to opinion leaders only applies under restrictive scope conditions that likely apply to few empirical scenarios. When diffusion follows the network strictly, as in the spread of a sexually transmitted disease (29) or clandestine communication with a cell structure, then centrality can have appreciable effects. However, the diffusion of a product, behavior, or belief, will normally involve some level of external influence, and even if that external influence is dwarfed by network influence, there should be no effect of the seed node’s network position so long as external influence exists at all.

Adding in even weak advertising effects nullifies the impact of seeding with the most central node. Advertising creates a nonzero probability that people can adopt without exposure from other adopters, conceptually similar to increasing the number of seeds. Our findings thus suggest that advertisers or public health officials who are planning a campaign should consider that advertising can also promote network-based spread and may do so more efficiently than identifying and recruiting a highly central seed node. This implies a return to the early “two-step flow” model, in which most people adopt based on influence from numerous minor opinion leaders of purely local influence, who in turn got information from mass media (19, 20).

There is substantial evidence that ideas and behaviors spread via interpersonal influence, but this is neither the same thing as an emergent property of critical importance for a highly central node nor a practical upshot that seeding with a central node is important under realistic circumstances. While social connections remain important for the spread of ideas, products, and behaviors, our simulations highlight the importance of the context in which those networks are embedded. Our results imply that in studies of diffusion the effect of mass media and advertising on the spread of a trend changes the nature of network-based diffusion, even if mass media and advertising have a weak role in and of themselves. To understand the drivers behind a trend, it is not sufficient to understand how well positioned the initial adopter is to spread the trend. We must also understand whether advertising or other broad forces like mass media, government mandates, or search engines seed the trend widely, and thereby render the choice of the initial adopter, no matter how central to the network, irrelevant.

## Supplementary Material

Supplementary File

## Data Availability

R code and data have been deposited in the Open Science Framework (10.17605/OSF.IO/25RAV).

## Footnotes

*Aside from measuring influence over diffusion, the other two major theoretical interpretations of network centrality are status and bargaining power (9, 10). These theoretical applications have their own associated centrality metrics.

^†^Replication code is available at https://osf.io/25rav/.

^‡^More formally stated, $P\left(X_{i}=t\text{|}X_{i}\geq t\right)=\alpha +\beta \left(\left(\sum_{j\in N\left(i\right)}X_{j}<t\right)/\left|N\left(i\right)\right|\right),$ where $X_{i}$ is the time when person *i* adopts, *N*(*i*) is the set of neighbors of person *i*, *α* is a constant hazard of adoption, representing the weight given to advertising and other external influences on diffusion, and *β* is the weight given to social or network influence.

^§^Parameter values are set to the same scale so that a one-unit change in *α* does not have a substantially different meaning than a one-unit change in *β*. We bracket the question of what position in parameter space is most realistic for what applications. However, neither the exact choice of maxima nor the exact position in parameter space matter for our findings, as the great bulk of the effect occurs with the introduction of any external influence.

^¶^See *SI Appendix, Multiple seeds* for specifications with multiple seeds targeted by key player (5). More seeds result in faster diffusion, but the relative advantage of targeting multiple seeds with key player versus an equal number of random seeds is an order of magnitude weaker than that of a single targeted seed versus a single random seed. However, the advantage of targeting multiple seeds is less fragile to the introduction of external influence.

^#^Centrality metrics tend to be correlated in the right tail, implying that the analysis should be robust to the choice of metric. As an example, *SI Appendix*, Fig. S7 replicates our findings using closeness centrality instead of betweenness.

^||^See *SI Appendix*, Fig. S1 for spaghetti plots of individual CDFs.
