# Supplementary material for: Network hubs cease to be influential in the presence of low levels of advertising
Source: Proc Natl Acad Sci U S A. 2021 Feb 12;118(7):e2013391118. doi: 10.1073/pnas.2013391118 (PMC7896329; doi:10.1073/pnas.2013391118)
Supplement: Supplementary File [file pnas.2013391118.sapp.pdf]

PNAS
www.pnas.org

Supplementary Information for

Network Hubs Cease to be Influential in the Presence of Low Levels of Advertising

Gabriel Rossman and Jacob C. Fischer

Gabriel Rossman
Email: rossman@soc.ucla.edu

This PDF file includes:

Supplementary text
Figures S1 to S12
Tables S1 to S3
SI References

Other supplementary materials for this manuscript include the following:

Replication files available at https://osf.io/25rav/wiki/home/

Simulation procedure

Agent based model

We develop an agent-based model version of the Bass(1) model of adoption. Our model takes the following steps.

1. Seed a network with a single initial adopter.
    - In some trials the seed node is chosen at random and in others the seed node is the highest centrality node in the network.
2. Calculate each person's probability of adopting at each discrete time interval using the following relationship: $P(X_i = t|X_i \geq t) = \alpha + \beta\left(\frac{\sum_{j \in N(i)} X_j < t}{|N(i)|}\right) = \alpha + \beta A Y_t$, where:
    - α and β are scalar parameters expressing external influence and network influence, respectively. For any given cell in parameter space they are fixed.
    - Y_t is an $n \times 1$ binary vector whose elements y_{it} are 1 if $X_i < t$, meaning that i adopted the innovation before time t, and 0 otherwise.
    - A is the $n \times n$ row-normalized adjacency matrix of the network, whose cells a_{ij} are defined:

      $$a_{ij} = z_{ij} / \sum_j z_{ij}$$

      where z_{ij} is 1 if i and j are connected and 0 otherwise.
3. Repeat step 2 until the network achieves the target level of saturation or until the maximum number of ticks has been completed.

We repeat this procedure for 1,000 trials for each cell in parameter space. For some networks, we expanded this to up to 5,000 trials to minimize right-censorship.

Network used in the experiments

Preferential attachment networks(2) -- generated networks in R using igraph::sample_pa(). The networks vary only by number of nodes and all of them have edges/node and power exponent set to one. This results in a tree-like structure. To measure key network traits, we generated a thousand networks of each type. We report the average of their mean path lengths and the betweenness score for the highest betweenness node in each network expressed as a Z-score.

Table S1

Number of generated networks	Nodes	Average mean path length	Peak betweenness nodes (Z-score)
1,000	1,000	8.4	18.7
1,000	10,000	11.5	58.6

Small world networks(3) -- generated in R using igraph::sample_smallworld(). We generated 1,000 examples of each type and varied number of nodes and edges/node. For these networks, the table below reports average mean path length and peak betweenness as a Z-score. The given parameters and measured attributes are summarized below.

Table S2

Number of generated networks	Parameters to generate the networks			Measured attributes of the networks	
	Nodes	edges/node	rewiring probability	average mean path length	Peak betweenness nodes (Z-score)
1,000	10,000	3	2%	12.4	10.8
1,000	1,000	2	2%	13.3	7.1
1,000	1,000	3	2%	8.5	6.8
1,000	1,000	4	2%	6.6	6.5

Empirical networks – We tested three empirical examples of real-world communication networks: the DNC e-mail network, the Enron e-mail network, and a Twitter network. The *Democratic National Committee (DNC) e-mail network* is the set of Democratic party e-mails posted to Wikileaks in 2016, available online at http://konect.uni-koblenz.de/networks/dnc-temporalGraph. The *Enron e-mail network* consists of Enron e-mails collected by the Federal Energy Regulatory Commission in 2002, available online at http://snap.stanford.edu/data/email-Enron.html. The *Twitter* network contains users who mention or retweet others during January 23 - February 8, 2011 and is available online at http://www-levich.engr.ccny.cuny.edu/~min/retweetformat.txt.(4) We forced DNC and Twitter to be undirected but Enron was already undirected. For each network we eliminated redundant edges and isolated the giant component. This last step biases the analysis in favor of the hypothesis that hubs are influential. For the two networks that

were originally directed, DNC and Twitter, we kept only nodes that both sent and received to identify the active core of the network before making them undirected. The attributes of these networks are summarized in the table.

Table S3

Network	Nodes	Edges	Mean path length	Peak betweenness node (Z-score)
DNC	548	2,442	2.9	17.2
Enron	33,696	180,811	4.0	70.7
Twitter	532,325	694,606	9.08	391.1

Determining parameter range

The α and β parameters are not inherently on the same scale. Indeed, any given value of α implies much more rapid diffusion than the same value for β. We use a simulation approach to rescale the parameters to be on the same scale. Under our approach, we allow parameters to range from 0 to the parameter value at which 50% of trials for a given network will be completely saturated by 100 ticks. We refer to this parameter value as the LD50, following terminology from toxicology for the median lethal dose, meaning the dose at which 50% of the test population perished.

Note that by setting comparable maxima for α and β we are not implying where the most realistic position is in parameter space and indeed this probably varies by empirical scenario.(5) For awareness, or learning about ideas or products, diffusion tends to be characterized by α. However, adoption, or actually embracing new ideas or purchasing new products, tends to skews towards β, especially when the new idea or behavior has low legitimacy or would be onerous to implement. There are exceptions though and adoption can skew towards α if there is aggressive promotion and the product being promoted is a good fit with the expectations of its target market.(6, 7)

We estimated these LD50 values using a separate set of simulations. We repeatedly run simulated diffusion processes on the networks that we use to determine the appropriate LD50 values through trial and error. We calculate LD50 values separately for each network formation heuristic or empirical network. For instance, preferential attachment with a thousand nodes has one set of LD50s, preferential attachment with ten thousand nodes another, and the Enron network a third.

In our main set of simulations, we use LD50 values to define the maximum for the range of each dimension of parameter space and we explore parameter space by taking 21 logarithmically spaced steps from 0 to the LD50 value for both α and β. We compute the logarithmically spaced percentage steps with the following R code:

```
expm1(seq(from = 0, to = log1p(100), length.out = 21)) / 100
```

and then multiply this vector by the LD50 value for each parameter of each network. This results in the following sequence:

```
0.0%, 0.3%, 0.6%, 1.0%, 1.5%, 2.2%, 3.0%, 4.0%, 5.3%, 7.0%, 9.0%,
11.7%, 14.9%, 19.1%, 24.3%, 30.9%, 39.1%, 49.5%, 62.7%, 79.2%, 100.0%
```

For example, DNC has an LD50 of 6.52 for α, which results in the following intervals (rounded to two digits for clarity):

```
0.00, 0.02, 0.04, 0.07, 0.10, 0.14, 0.20, 0.26, 0.35, 0.46, 0.59, 0.76,
0.97, 1.24, 1.58, 2.01, 2.55, 3.23, 4.09, 5.16, 6.52
```

Where 0.02 = 6.52 * 0.003, 0.04 = 6.52 * 0.006, etc.

DNC's LD50 of 27.98 for β results in:

```
0.00, 0.07  0.16  0.28  0.42  0.61, 0.84, 1.13, 1.49, 1.95, 2.53, 3.26,
4.18, 5.34, 6.80, 8.63, 10.95, 13.86, 17.53, 22.16, 27.98
```

R code for this process would be as follows:

```
alpha_ld50 <- 6.52
beta_ld50 <- 27.98
log_pcts <- expm1(seq(from = 0, to = log1p(100), length.out = 21)) /
100
alpha_intervals <- alpha_ld50 * log_pcts
beta_intervals <- beta_ld50 * log_pcts
```

At a conceptual level, we are taking logarithmically spaced steps of percentages then we multiply those logarithmically spaced steps by the relevant LD50 to establish the intervals. This gives us a fine-grained view of low values, which is important as the collapse of a special role for high centrality nodes occurs at very low values for α; typically below 1% of α's LD50. We prefer the order of operations in which we take the logs of percentages then multiply by the LD50 over simply taking the logarithms of LD50s in that it results in scale intervals that are intuitively comparable regardless of the maximum. Since β LD50s are always greater than α LD50s, our order of operations biases the analysis against our hypothesis that low levels of α qualitatively change the nature of diffusion.

Right censorship

When both parameters were set to extremely low values, the simulation can take a very long time to reach mid-saturation in all 1,000 trials per cell. We allowed simulations to run for either 1,000 iterations (Twitter, preferential attachment with 10,000 nodes, and all small world networks) or 5,000 iterations (DNC email, Enron email, and preferential attachment with 1,000 nodes). Nonetheless, some cells still had right-censored trials. If less than 10% of trials in a cell were right-censored, we top-coded those right-censored cells at the maximum number of iterations the trial was allowed to run. If more than 10% of trials in a cell were right-censored, we treated that cell as missing. The level of missing data and the top-code for each treatment condition in each cell of parameter space can be found in the replication files ending ".rds" as the columns *missing_reps* and *max.ticks*, respectively. This top-coding has the effect of biasing the results slightly towards parity between experimental conditions, especially in the region of parameter space where $\alpha = 0$ and β is close to 0. Since right-censorship is almost entirely an issue for cells where

$\alpha = 0$, top-coding has the effect of making the plane where $\alpha = 0$ more similar to the rest of parameter space. This biases our results *against* our argument that opinion leadership is distinct to $\alpha = 0$.

Readers who prefer to see the results with differing tolerance for right-censored data may use the replication files and change the parameter *p_censored_ok* in the main RMarkdown file. For instance, to drop cells entirely that have any right-censored trials, please set *p_censored_ok* <- *0*. (Note that Twitter has some missing data for every cell in the crucial left column and so dropping *p_censored_ok* < .03 will drop the left column of the heat map with the result that the scale is radically compressed and the heat map will display noise among substantively similar cases.)

CDF plots of $\alpha = 0, \beta = LD50$ *and* $\alpha = 0.0026LD50, \beta = LD50$ *for random and high betweenness seeds*

Figure 3 illustrates that the key transition in parameter space occurs when medium to high network influence (β) combines with either zero or a tiny bit of external influence (α). In Figure S1, we visualize the micro-dynamics of the rapid decay of the importance of central nodes with spaghetti plots showing individual CDFs with ribbon plots showing the central tendencies. Note that the top panels are a minor variation on figure 2 but the introduction of the spaghetti plots shows that the principal difference between random and high betweenness seeds is not how fast they can be, but how slow, with the fastest CDFs being similar, but random seeds having many glacial CDFs.

The bottom panels illustrate three things about the micro-level effect of introducing a modicum of external influence. First, as would be expected from the corresponding cell in the heat map, the two bottom panels are much more similar than are the top two panels. Second, the bottom-left panel (displaying randomly seeded simulations with low external influence) has CDFs that are still qualitatively s-curves, validating that this point in parameter space really is primarily an endogenous process and the external influence at this level is mostly a catalyst to change the nature of endogenous diffusion rather than an appreciable diffusion force itself. Third, the introduction of a small external influence makes the individual CDFs less stochastic and in particular nearly eliminates laggard outliers. In contrast, when $\alpha = 0$, $\beta = LD50$ (top panels), there is enormous variation in diffusion trajectories, especially with a random seed node. At the micro-level, a small amount of external influence not only makes diffusion faster, it makes it more predictable.

This is all illustrated with a difference between α set to zero versus about one quarter of a percent of LD50 and even that tiny increment shows visually striking differences. At slightly larger levels of α of 1% or 2% of LD50, the CDFs based on random vs. high betweenness seeds are indistinguishable.

Figure S1

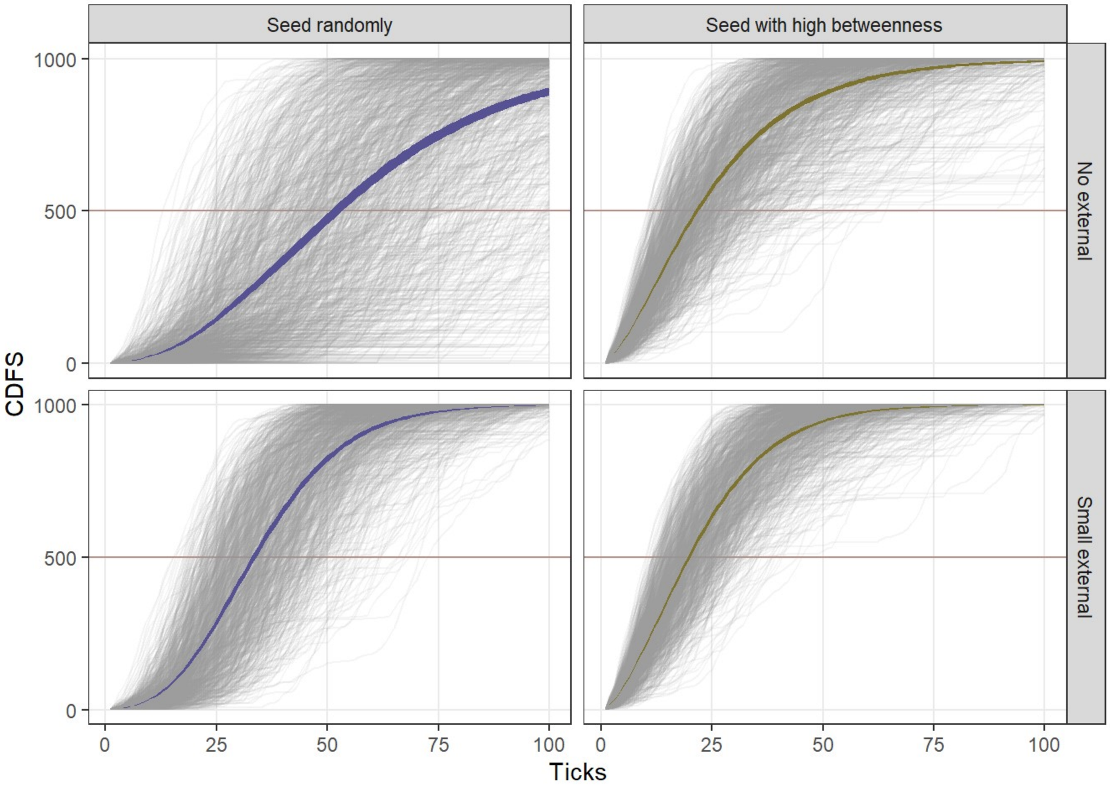

CDFs for diffusion on a preferential attachment network with a thousand nodes. The mean trajectory with confidence intervals for each panel is shown as a ribbon plot. Individual trials are shown as spaghetti plots. The panels represent α = 0, β=LD50 (top) versus $\alpha = 0.26\% \times LD50, \beta = LD50$ (bottom) and random seeds (left) vs high betweenness seeds (right).

Correspondence of Figure 2 to Figure 3

Figure S2

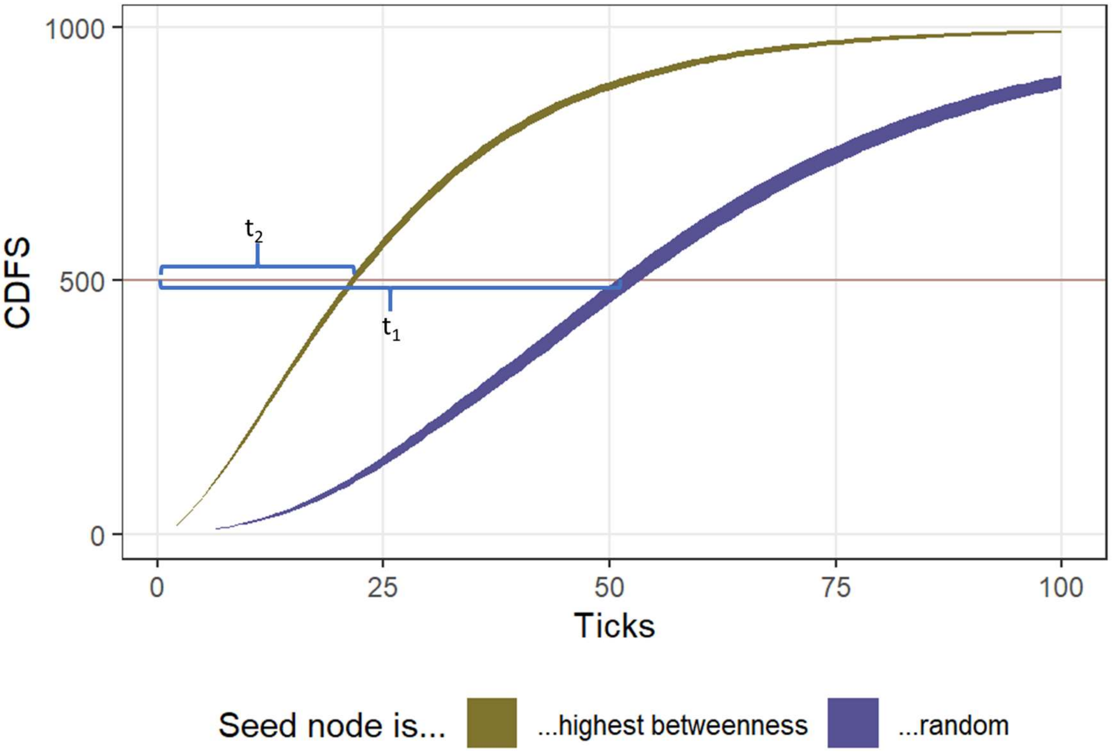

Same as Figure 2 but annotated with brackets to highlight the mean time to mid-saturation for randomly seeded trials (t_1) and trials seeded with the highest centrality node (t2). The heat dimension in all heat maps (e.g., the top panel of figure 3) represents the ratio of t_1:t_2. Likewise, the height of the sparklines in the bottom panel of figure 3b represents the ratio of t_1:t_2.

Preferential attachment networks

In Figure S3, we reproduce the top panel of figure 3 (based on 1,000 node preferential attachment networks) and replicate it for networks with 10,000 nodes.

Figure S3

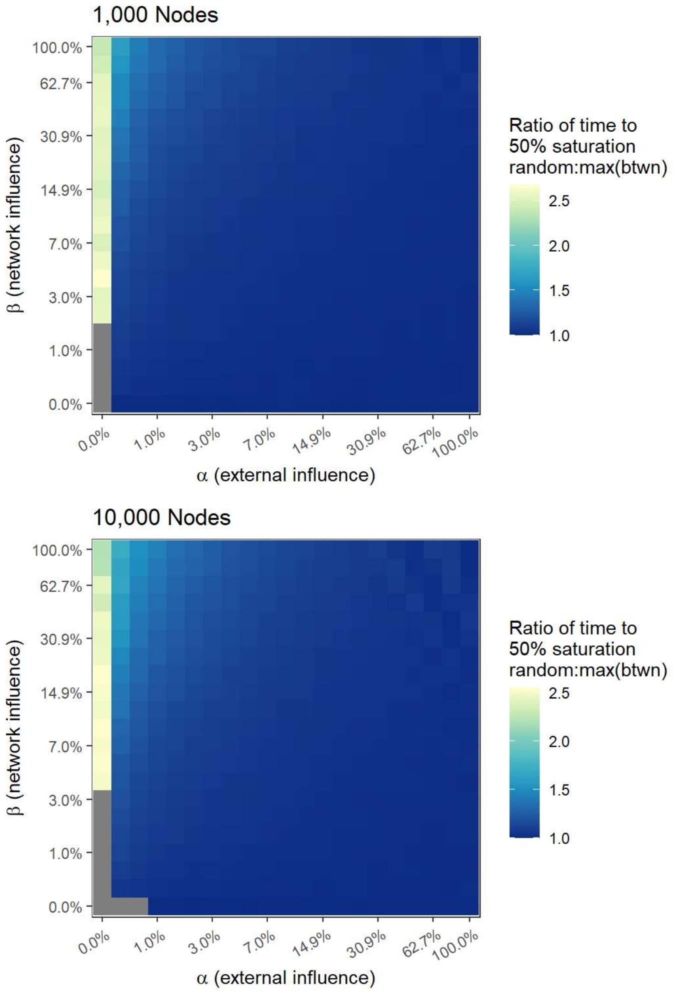

Heatmap of ratio of time to adoption. Gray cells represent right censored cases after 5,000 iterations (1,000 nodes) or 1,000 iterations (10,000 nodes). Ratio of time to adoption in simulations targeting the highest betweenness node versus targeting a randomly chosen node in the network. The sampled graphs follow a preferential attachment network (either 1,000 or 10,000 nodes, 1 edge/node, and an exponent of 1).

Small world networks

In Figures S4 and S5, we rerun the simulation on Watts-Strogatz small world networks. A small number of generated small world networks had disconnected components. We replaced these networks with newly generated networks to bias the simulation in favor of opinion leadership. Aside from the different network (small world vs. preferential attachment), the figures are similar to the top panel of Figure 3.

Figure S4

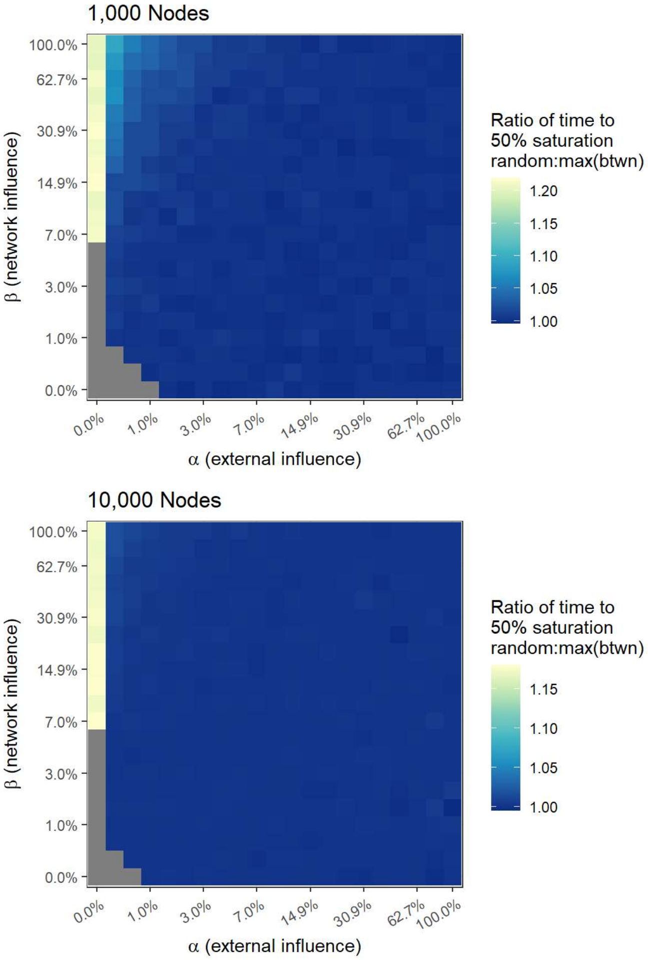

Heatmap of ratio of time to adoption. Gray cells represent right censored cases after 1,000 iterations. Ratio of time to adoption in simulations targeting the highest betweenness node versus targeting a randomly chosen node in the network. The sampled graphs follow a Watts-Strogatz small world network (either 1,000 or 10,000 nodes, 3 edges/node, and 2% probability of random rewiring).

Figure S5

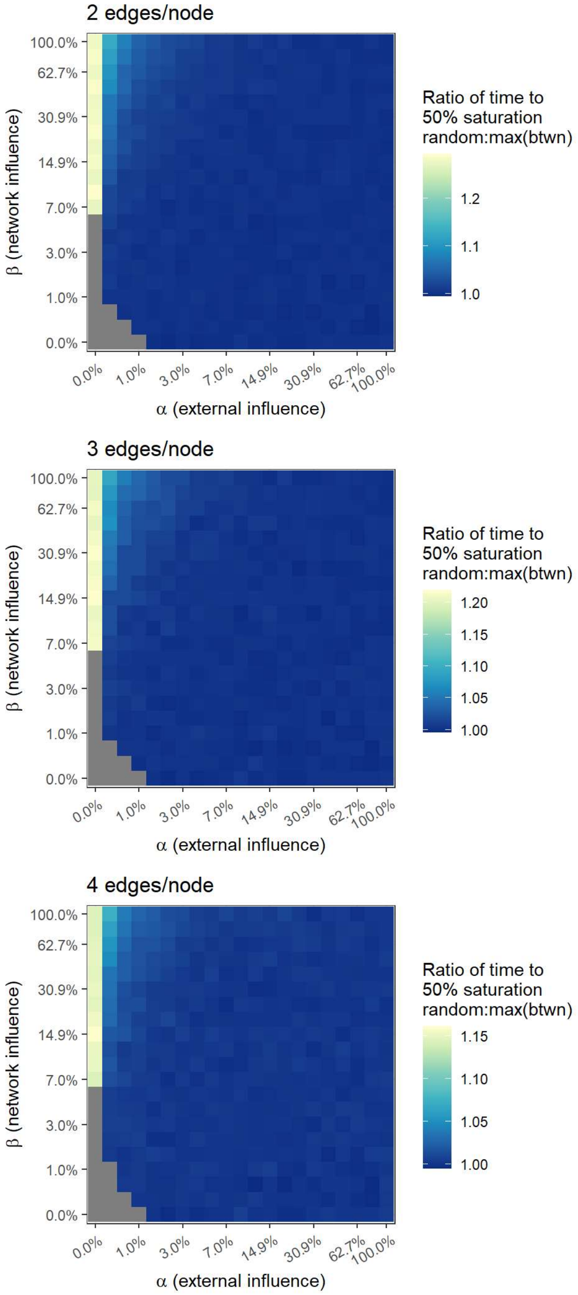

Heatmap of ratio of time to adoption. Gray cells represent right censored cases after 1,000 iterations. Ratio of time to adoption in simulations targeting the highest betweenness node versus targeting a randomly chosen node in the network. The sampled graphs follow a Watts-Strogatz small world network (1,000 nodes, 2-4 edges/node, and 2% probability of random rewiring).

Empirical networks

In Figure S6, we rerun the simulation using three empirical networks. The specification is otherwise similar to the top panel of Figure 3.

Figure S6

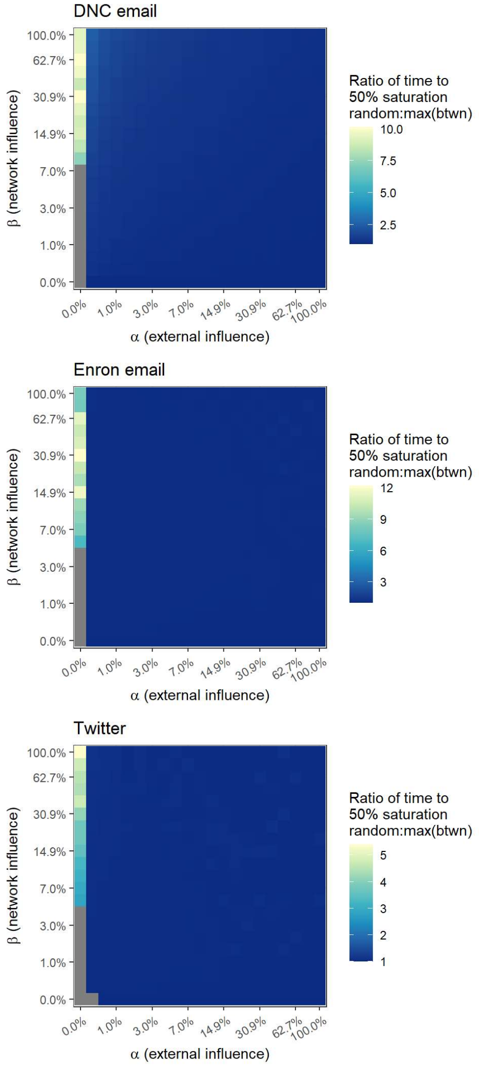

Heatmap of ratio of time to adoption. Gray cells represent points in parameter space where over 10% of trials were right censored after 5000 iterations (DNC and Enron) or 1000 iterations (Twitter). For cells with less than 10% right-censorship, right-censored trials were top-coded at 1000 iterations (Twitter) or 5000 iterations (DNC and Enron).

Closeness centrality

We primarily use betweenness centrality to identify seed nodes, but in this alternate specification (figure S7) we experiment with closeness centrality as a demonstration that our findings are not limited to using betweenness to identify seed nodes.

Figure S7

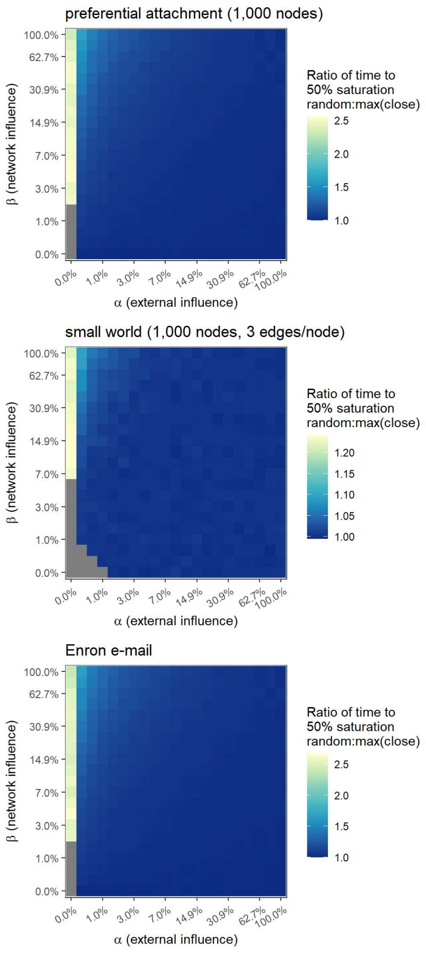

Heatmap of ratio of time to adoption comparing simulations seeded with the highest closeness node versus seeded at random. Gray cells represent >10% right censored cases after 1,000 iterations (small world) or 5,000 iterations (preferential attachment and Enron). Aside from using closeness centrality to choose the seed node, the specification is identical to that in Figures 3 (top panel), S5 (middle panel), and S6 (middle panel).

Multiple seeds

We primarily use a single seed node. In figures S8, S9, and S10, we compare this to 1% seeds and 5% seeds. Figure S8 shows the ratio of mid-saturation diffusion times for random versus targeted nodes when $\beta = LD50$ and α varies along the usual log scale between 0 and LD50. LD50 values are re-estimated for 1% and 5% seeds. Figures S9 and S10 show heat maps for preferential attachment graphs (1,000 nodes) and DNC E-mail (548 nodes), respectively.

Because betweenness centrality scores a node's importance to the network irrespective of whatever nodes have already been identified, it is not well suited for identifying multiple seeds. We thus identify multiple seeds using Borgatti's keyplayer algorithm as implemented in the R library influenceR to identify a 5% seed.(8, 9).

The multiple seed results differ in two notable ways from results based on a single node. First, as shown in Figure S8, the baseline effect of targeted seeding in the absence of external influence is much smaller. In the region of parameter space that is ideal for opinion leadership ($\alpha = 0, \beta = LD50$), a single random seed takes 150% longer than a maximum betweenness seed to saturate half of a preferential attachment network and 881% longer to saturate the DNC e-mail network. In contrast, a 1% random seed versus a 1% seed identified by the keyplayer algorithm takes 15% longer to saturate half of a preferential attachment network and 47% for DNC. The comparable figures for a 5% seed are 8% on a preferential attachment and 15% longer on DNC e-mail. Thus, choosing an optimal 5% seed is at least an order of magnitude less important than choosing an optimal single seed.

Second, as shown in figures S9 and S10, the drop-off in the advantage of a highly targeted seed diminishes with multiple seeds. This is fairly minimal with a 1% seed as both preferential attachment and DNC e-mail still show something like the yellow stripe and blue field pattern familiar from this paper's other heat maps. The only difference is that the 1% seed heat maps show a gradient for values of $\alpha < 0.03 \times LD50$. Nonetheless, the 1% seed heat maps mostly resemble the single seed heat maps. The gradient extends much further with a 5% seed and now appears smooth. Note that the heat map has a log-log scale, such that even with a 5% seed, the baseline advantage ($\alpha = 0, \beta = LD50$) nearly disappears by the time $\alpha > 0.3 \times LD50$. However, this should not be discounted as this is a plausible region of parameter space for many empirical applications.

We must thus conclude that the effect of targeting multiple seeds is much smaller but also more robust to the introduction of external influence than is targeting a single seed and that both the decline in the baseline effect and the robustness to the introduction of external influence are roughly proportional to the size of the seed. Under many realistic scenarios, targeting multiple seeds may be practical so long as the cost of identifying and targeting specific seeds is low and external influence is very expensive and/or very ineffective. Outlining the contours of this trade-off is an issue for future research.

Figure S8

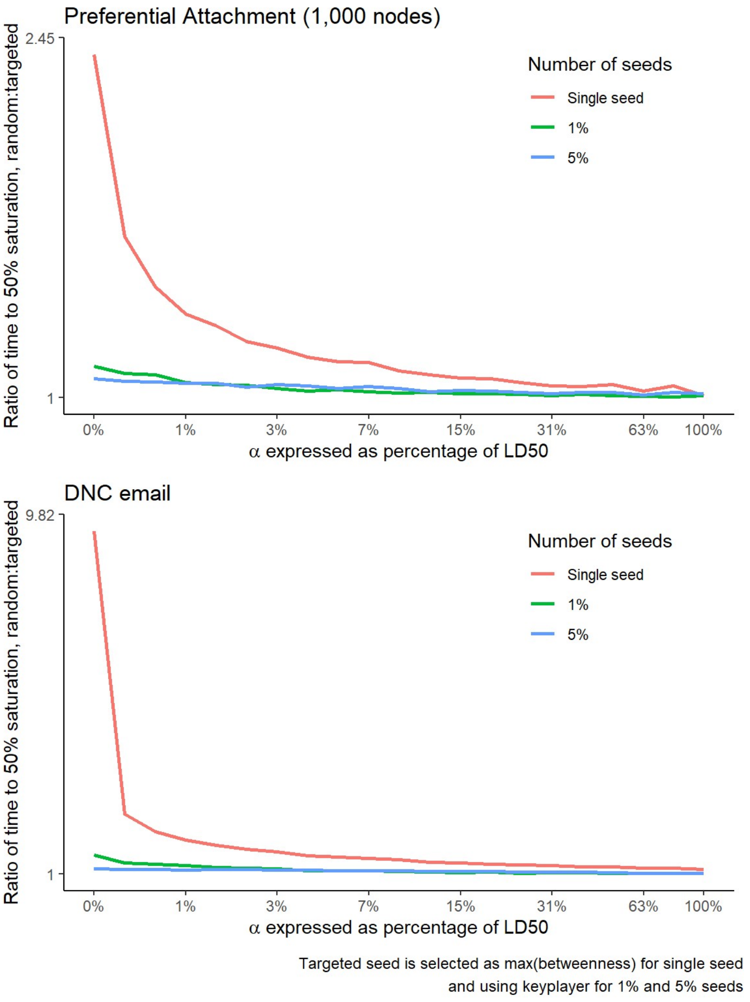

Line graph of ratio of time to adoption comparing simulations on 1,000 node preferential attachment networks (top panel) or DNC email network (bottom panel) with random seed(s) to targeted seed(s) identified by maximum betweenness for a single seed or keyplayer for 1% or 5% seeds. Both panels assume high levels of network diffusion ($\beta = LD50$) but vary external influence (α) as a percentage of each LD50 value, plotted on a logarithmic scale. This is the equivalent to the top row of cells in Figures S9 and S10 but substituting a y-axis for the heat dimension. In both preferential attachment and DNC, the baseline effect of targeting a single node is higher but more sensitive to rising α.

Figure S9

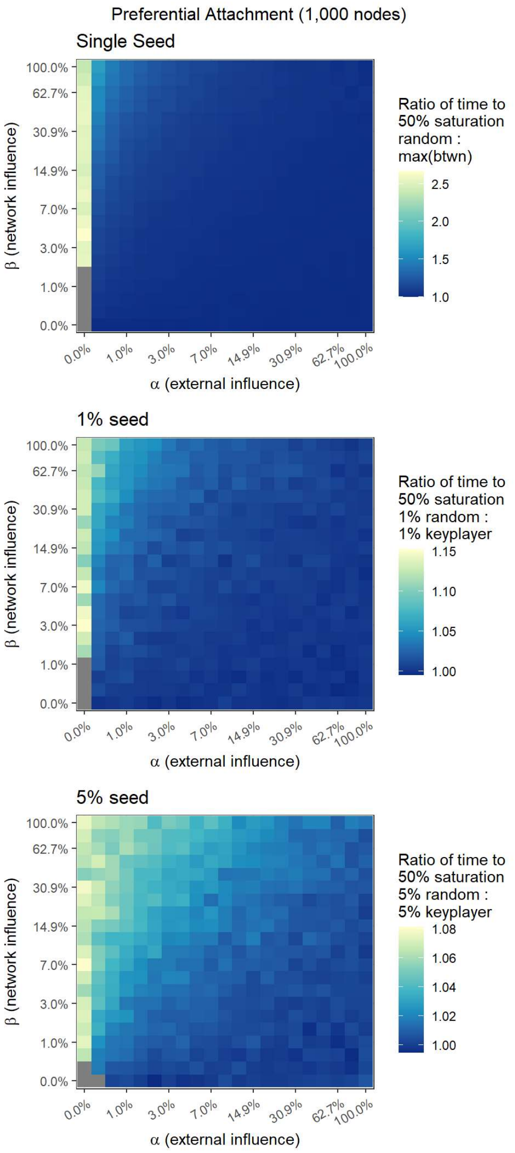

Heat maps summarizing ratio of time to adoption for random seed(s) versus targeted seed(s) on preferential attachment networks with 1,000 nodes. Targeted seeds are identified by maximum betweenness for a single seed or keyplayer for 1% or 5% seeds. Gray cells represent >10% right censored cases after 5,000 iterations.

Figure S10

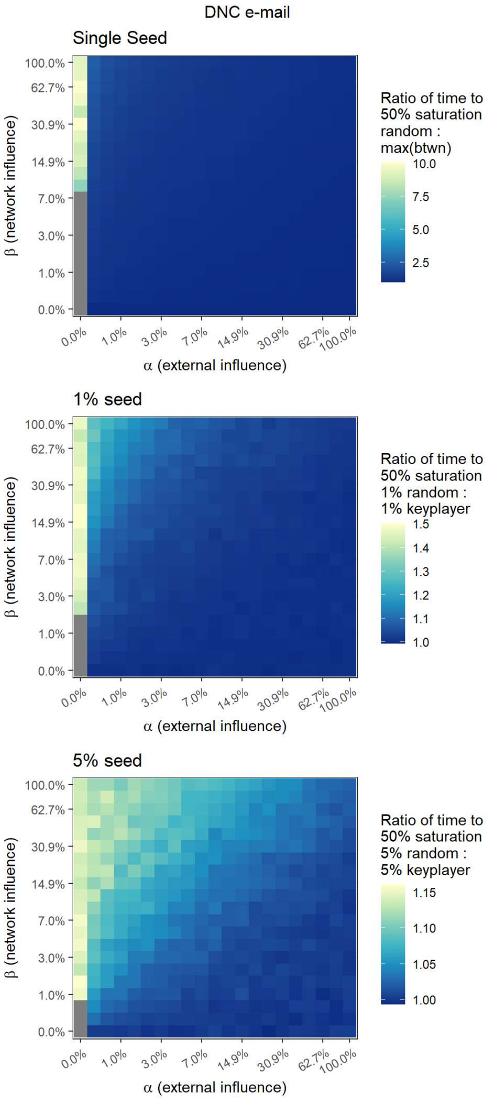

Heat maps summarizing ratio of time to adoption for random seed(s) versus targeted seed(s) on DNC email network. Targeted seeds are identified by maximum betweenness for a single seed or keyplayer for 1% or 5% seeds. Gray cells represent >10% right censored cases after 5,000 iterations.

Status-biased Diffusion

In most of our models we treat network-based diffusion pressure as proportional to the percentage of a node's alters who have already adopted. In Figure S11 we allow high degree nodes to have more influence. Theoretically this reflects a heuristic that nodes see their high degree neighbors as prestigious and especially worthy of emulation. In the status-biased model, the baseline effect $(\alpha = 0, \beta = LD50)$ is slightly stronger than in the standard model (midpoint saturation is 3.3x vs 2.7x faster with a hub seed). However, both models show this paper's general pattern that the advantage for seeding with the most central node disappears with the introduction of any external influence.
A quick overview of status-biased diffusion in our model.
Take the following network:

$$A = \begin{bmatrix} 0 & 1 & 1 & 1 & 0 \\ 1 & 0 & 0 & 0 & 0 \\ 1 & 0 & 0 & 0 & 1 \\ 1 & 0 & 0 & 0 & 0 \\ 0 & 0 & 1 & 0 & 0 \end{bmatrix}$$

Normally we row normalize and multiply by a column vector to get the percentage of one's alters who have adopted. Concretely, let's say person 1 (row / column 1) is the only adopter. We would do the following:

$$\begin{bmatrix} 0 & 1/3 & 1/3 & 1/3 & 0 \\ 1 & 0 & 0 & 0 & 0 \\ 1/2 & 0 & 0 & 0 & 1/2 \\ 1 & 0 & 0 & 0 & 0 \\ 0 & 0 & 1 & 0 & 0 \end{bmatrix} \begin{bmatrix} 1 \\ 0 \\ 0 \\ 0 \\ 0 \end{bmatrix} = \begin{bmatrix} 0 \\ 1 \\ 1/2 \\ 1 \\ 0 \end{bmatrix}$$

Adoption is then proportional to the resulting column vector.
For figure S11, we bias in favor of high status people by multiplying each column by a function of its sum (the person's degree) before row normalizing. (Namely, we multiply but the log of degree in accordance with our intuition that one's status bias increases with the order of magnitude of one's popularity.) For example, the columns multiplied by their sums would give:

$$\begin{bmatrix} 0 & 1 & 2 & 1 & 0 \\ 3 & 0 & 0 & 0 & 0 \\ 3 & 0 & 0 & 0 & 1 \\ 3 & 0 & 0 & 0 & 0 \\ 0 & 0 & 2 & 0 & 0 \end{bmatrix}$$

And the row-normalized multiplication would be:

$$\begin{bmatrix} 0 & 1/4 & 1/2 & 1/4 & 0 \\ 1 & 0 & 0 & 0 & 0 \\ 3/4 & 0 & 0 & 0 & 1/4 \\ 1 & 0 & 0 & 0 & 0 \\ 0 & 0 & 1 & 0 & 0 \end{bmatrix} \begin{bmatrix} 1 \\ 0 \\ 0 \\ 0 \\ 0 \end{bmatrix} = \begin{bmatrix} 0 \\ 1 \\ 3/4 \\ 1 \\ 0 \end{bmatrix}$$

This is equivalent to taking the weighted percentage of one's alters who have adopted, where the weights are given by the degree. If the degree of the i-th node is d_i, then the weighted percentage of alters is

$$\left(\frac{d_i}{\sum_{i=1}^{n} d_i}\right)\mathbf{1}(i \text{ adopted})$$

When unweighted, $d_i = 1$ for all i, and this simplifies to

$$\left(\frac{1}{n}\right)\mathbf{1}(i \text{ adopted})$$

Figure S11

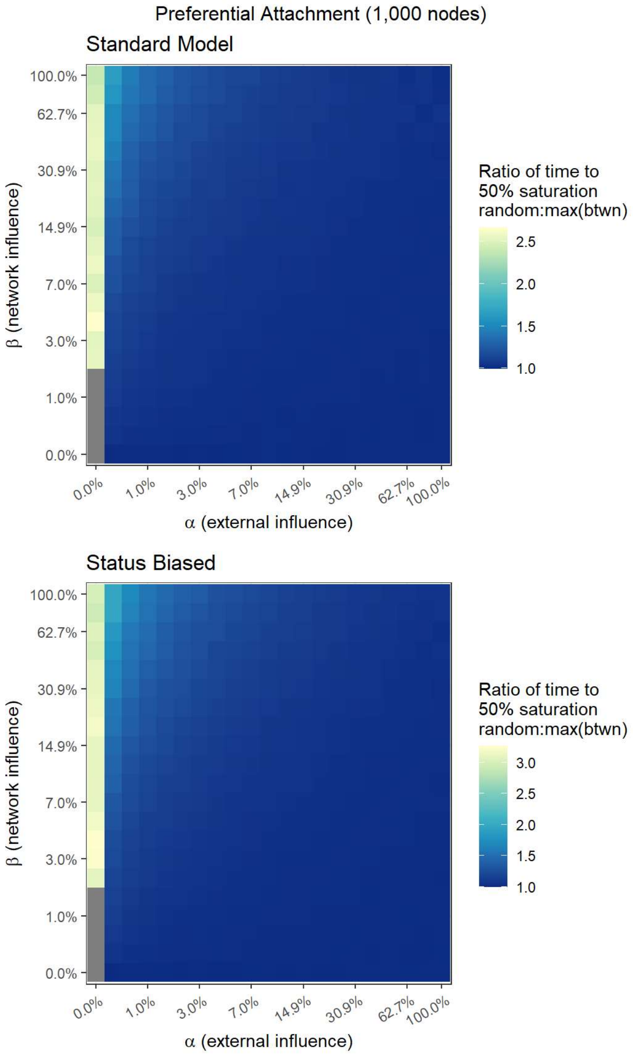

Heat maps summarizing ratio of time to adoption for random seed(s) versus targeted seed(s) on preferential attachment network. The top panel assumes nodes are equally influential and the bottom panel assumes high degree nodes are more influential. Gray cells represent >10% right censored cases after 5,000 iterations.

Heterogeneity of Susceptibility to External Influence

In most of our simulations, we assume that all nodes are equally susceptible to external influence. However, we can imagine that some people are invulnerable to external influence. Perhaps they do not see television advertisements as they either do not own a television or only watch Netflix. Or in the case of government mandates, maybe some firms are exempt from the mandate by virtue of being below a certain threshold of employees or having no government contracts. (The employee threshold is particularly important in France and reliance on government contracts in the United States).(10, 11) Or maybe some people are simply intensely skeptical of external influence.

To account for these possibilities, in Figure S12 we relax the assumption of equal susceptibility to external influence by allowing for the possibility that 20% or 50% of nodes completely ignore external influence. Note that under this assumption the LD50 for α is undefined, however in a qualitative sense the LD50 is still a reasonable approximation (albeit a conservative one) so long as the fraction of nodes invulnerable to external influence is not too high.

The results with 20% of nodes being invulnerable to external influence are indistinguishable from those where all nodes are susceptible to external influence. The results with 50% of nodes being invulnerable are also broadly similar but have two properties of note. First, the bottom row of the heat map where $\alpha = 0$ is undefined. This is necessarily true as the measure is based on how many time periods it takes for adoptions to exceed 50% and this is impossible for these positions in parameter space. Second, there is a slightly longer gradient than usual as the effect of highly central seeds takes a bit higher values of α to fully fade out. However, this is easily explicable by the LD50 being undefined and us substituting a value that in a qualitative sense is probably about half as big as it ought to be. The model is remarkably robust to a small to moderate number of nodes being completely invulnerable to external influence. This reflects the classic two-step flow model where many people are completely inattentive to mass media on a particular subject but learn about new products, ideas, and behaviors from locally influential opinion leaders who themselves are attentive to mass media.(12)

Figure S12

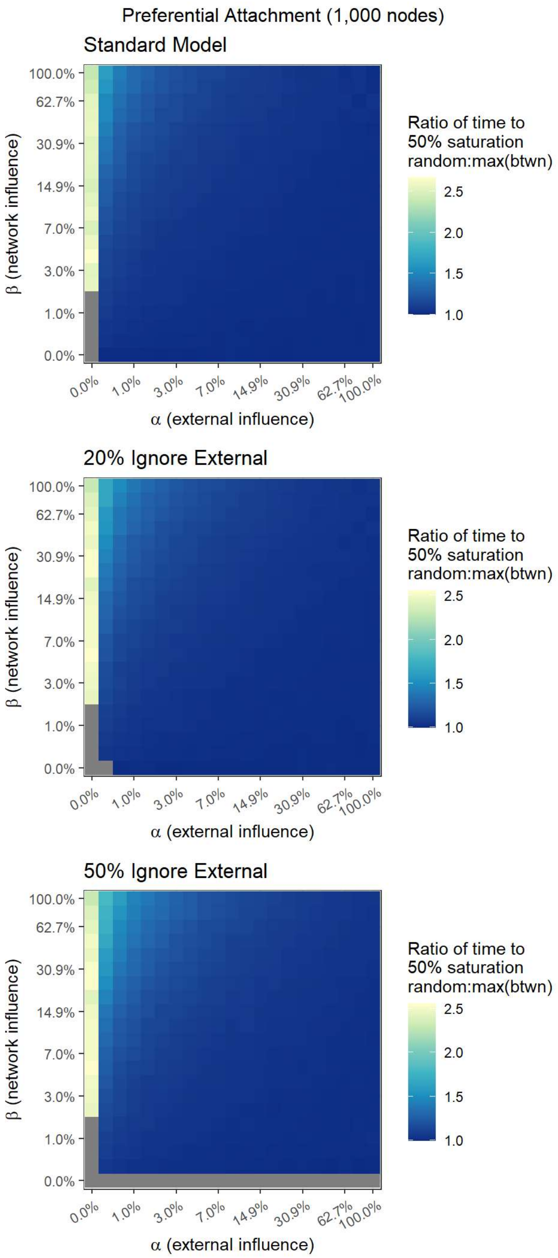

Heat maps summarizing ratio of time to adoption for random seed(s) versus targeted seed(s) on preferential attachment network. The top panel is the standard model. The middle panel and bottom panel show results when 20% or 50%, respectively, of nodes completely ignore external influence. Gray cells represent >10% right censored cases after 5,000 iterations.

SI References

1. F. M. Bass, A New Product Growth for Model Consumer Durables. *Manag. Sci.* **15**, 215--227 (1969).
2. A.-L. Barabási, R. Albert, Emergence of Scaling in Random Networks. *Science* **286**, 509–512 (1999).
3. D. J. Watts, S. H. Strogatz, Collective Dynamics of "Small-World" Networks. *Nature* **393**, 440–442 (1998).
4. F. Morone, H. A. Makse, Influence maximization in complex networks through optimal percolation. *Nature* **524**, 65–68 (2015).
5. T. W. Valente, Diffusion of Innovations and Policy Decision-Making. *J. Commun.* **43**, 30–45 (1993).
6. G. Rossman, *Climbing the Charts* (Princeton University Press, 2012).
7. C. van den Bulte, G. L. Lilien, Medical Innovation Revisited: Social Contagion versus Marketing Effort. *Am. J. Sociol.* **106**, 1409–1435 (2001).
8. S. P. Borgatti, Identifying sets of key players in a social network. *Comput. Math. Organ. Theory* **12**, 21–34 (2006).
9. S. Jacobs, A. Khanna, *influenceR: Software tools to quantify structural importance of nodes in a network* (2015).
10. F. Dobbin, F. R. Sutton, The Strength of a Weak State: The Rights Revolution and the Rise of Human Resources Management Divisions. *Am. J. Sociol.* **104**, 441–476 (1998).
11. L. Garicano, C. Lelarge, J. Van Reenen, Firm Size Distortions and the Productivity Distribution: Evidence from France. *Am. Econ. Rev.* **106**, 3439–3479 (2016).
12. E. Katz, P. Lazarsfeld, *Personal Influence: The Part Played by People in the Flow of Mass Communications* (Free Press, 1955).
